# HDAC inhibitor-induced drug resistance involving ATP-binding cassette transporters (Review)

**DOI:** 10.3892/ol.2014.2714

**Published:** 2014-11-19

**Authors:** XUAN NI, LI LI, GUOYU PAN

**Affiliations:** 1 Center for Drug Safety Evaluation and Research, Shanghai Institute of Materia Medica, Chinese Academy of Sciences, Shanghai 201203, P.R. China; 2 Office of Clinical Pharmacology, Center for Drug Evaluation and Research, US Food and Drug Administration, Silver Spring, MD 20993-0002, USA

**Keywords:** histone deacetylase inhibitors, cancer treatment, multidrug resistance, P-glycoprotein, breast cancer resistance protein, multidrug resistance protein 1/2, nuclear receptor

## Abstract

Histone deacetylase (HDAC) inhibitors are becoming a novel and promising class of antineoplastic agents that have been used for cancer therapy in the clinic. Two HDAC inhibitors, vorinostat and romidepsin, have been approved by the Food and Drug Administration to treat T-cell lymphoma. Nevertheless, similar to common anticancer drugs, HDAC inhibitors have been found to induce multidrug resistance (MDR), which is an obstacle for the success of chemotherapy. The most common cause of MDR is considered to be the increased expression of adenosine triphosphate binding cassette (ABC) transporters. Numerous studies have identified that the upregulation of ABC transporters is often observed following treatment with HDAC inhibitors, particularly the increased expression of P-glycoprotein, which leads to drug efflux, reduces intracellular drug concentration and induces MDR. The present review summarizes the key ABC transporters involved in MDR following various HDAC inhibitor treatments in a range of cancer cell lines and also explored the potential mechanisms that result in MDR, including the effect of nuclear receptors, which are the upstream regulatory factors of ABC transporters.

## 1. Introduction

Post-translational modifications to the histones of chromatin play an important role in the regulation of gene expression. One histone modification is histone acetylation, which is regulated by histone acetyl transferases (HATs) and histone deacetylases (HDACs) (1,2). Abnormal expression of these enzymes can contribute to carcinogenesis. Numerous studies have suggested that HDAC inhibitors are becoming an effective anticancer drug in cell lines and animal models of carcinogenesis (1,3,4). Currently, >20 HDAC inhibitors are undergoing preclinical and clinical tests, particularly vorinostat (Zolinza) and depsipeptide (Istodax), having been approved by the Food and Drug Administration (FDA) to be used for the treatment of T-cell lymphoma (5,6). Although HDAC inhibitors are potent anticancer agents, certain studies have revealed that HDAC inhibitors can induce multidrug resistance (MDR), contributing to a poor prognosis in cancer treatment (7,8). Numerous studies have demonstrated that the upregulation of adenosine triphosphate-binding cassette (ABC) transporters is the most common cause of MDR. Well-known ABC transporters include P-glycoprotein (P-gp), breast cancer resistance protein (BCRP) and MDR protein (MRP) (9). Among the ABC transporters, P-gp is the most common transporter to be responsible for the phenomenon of MDR (10,11).

The aim of the present review is to report the ABC transporters that are involved in MDR following various HDAC inhibitor treatments in a range of cancer cell lines, particularly emphasizing the upregulation of P-gp and suggesting a careful use of HDAC inhibitors in clinical treatment. Furthermore, the present review also intends to explore the possible mechanisms behind the issue of MDR, including the effect of nuclear receptors.

## 2. HDACs

Cancer has been hypothesized to originate from the abnormal expression of tumor suppressor genes or oncogenes, which always involves genetic alterations, including mutations, deletions, rearrangements and amplifications (12). Hanahan and Weinberg described the six most important factors for the development and progression of cancer, including the self-sufficiency in growth signals, the blocking of apoptosis and differentiation, and the stimulation of angiogenesis, proliferation and metastasis (13). These factors are regulated by epigenetic mechanisms that include non-histone and histone acetylation, which play a key role in tumorigenesis (12,14). The reversible acetylation is mainly controlled by HAT and HDAC (1,2,15). HATs catalyze the transfer of an acetyl group to lysine and lead to the activation of DNA transcription. HDACs deacetylate lysine residues, resulting in a closed chromatin conformation and repression of transcription (6). These changes are reversible in contrast to permanent gene mutations (12).

There are currently 18 human HDACs that have been identified based on their function and the phylogenetic analysis of their primary structures and homology to yeast enzymes (6,16). HDACs are divided into four classes: Class I, consisting of HDAC 1–3 and 8; class II, with class IIa consisting of HDACs 4, 5, 7 and 9, and class IIb consisting of HDAC 6 and 10; and class IV, which are all Zn^2+^-dependent, while class III HDACs use NAD^+^ instead of Zn^2+^ as their cofactor (14,17).

Aberrant protein acetylation, particularly of histones, has been associated with abnormal development and cancer. HDACs control numerous essential mechanisms during cell development and tissue maintenance. However, abnormal expression of HDACs has been found in a broad range of cancer types. In humans, certain studies have revealed the overexpression of HDAC1 in solid tumors, including gastric, colon, breast, pancreatic, hepatocellular, lung and prostate carcinomas (2,18). Other class I HDACs were also found to be highly expressed in the nuclei of a considerable number of colorectal carcinomas (18). Weichert *et al* found that HDAC expression was the highest in proliferating tumors (19). In addition to solid tumors, altered HDAC expression or mistargeted HDAC activity results in hematological malignancies, including lymphoma, leukemia and myeloma (12). Notably, numerous clinical studies have established that overexpression is the most common alteration of HDAC function in the tumors of patients with cancer (12). Therefore, preventing the aberrant function of HDACs by affecting HDAC expression, particularly overexpression, can be an attractive target for cancer therapy (2).

## 3. HDAC inhibitors

It is not unexpected that HDACs represent potential drug targets for cancer treatment. HDAC inhibitor-mediated acetylation results in changes in gene expression and the functional modification of histone and non-histone proteins, thereby triggering antitumor pathways. Inhibiting the overexpression of HDACs in particular can prevent tumorigenesis (12). An escalation in drug identification efforts has resulted in the development of HDAC inhibitors, a number of which have been pre-clinically revealed to possess potent anti-tumor activity. Several of these are already undergoing clinical trials, including vorinostat as a treatment for cutaneous T-cell lymphoma (CTCL) and romidepsin as a treatment for peripheral T-cell lymphoma (18,20).

The HDAC inhibitors that are being developed for cancer treatments can be divided into four chemical classes: cyclic tetrapeptides, including depsipeptide, apicidin and trapoxin; the hydroxamic acids, including suberoylanilide hydroxamic acid (SAHA), scriptaid, trichostatin A (TSA), pyroxamide and oxamflatin; short-chain fatty acids, including valproic acid (VPA), phenylbutyrate and butyrate (BT); and benzamides, such as MS-275 (21). Despite distinct chemical structures, these HDAC inhibitors exhibit similar mechanisms of action (15,22). These compounds mainly exert their inhibitory effect via the Zn^2+^ dependency of HDAC enzymes. HDAC inhibitors affect cancer cells by cell-cycle arrest, by promoting differentiation or apoptosis, and by affecting angiogenesis and the immune system through upregulation of tumor antigens (6,12,23). HDAC inhibitors, including TSA, vorinostat and panobinostat, contain a pharmacophore that includes a cap, connecting unit, linker and a zinc-binding group that chelates the cation in the catalytic domain of the target HDAC (23).

Numerous studies have been conducted for HDAC inhibitors, the majority of which focused on the mechanism of HDAC inhibitors as antineoplastic drugs, particularly on the ability of HDAC inhibitors to reactivate those genes involved in differentiation, cell cycle regulation, apoptosis, angiogenesis, invasion and metastasis (12,14,15,18,21). However, only certain studies focused on the poor prognosis following treatment with HDAC inhibitors, which could result in MDR of cancer cells when used alone or in combination with other chemotherapeutic agents (24–26). Clarification of the mechanism through which MDR originates following the use of HDAC inhibitors is required.

## 4. HDAC inhibitors and ABC transporters

The interest in HDAC inhibitors as potent anticancer drugs is due to their broad anti-tumor activity and low toxicity in normal cells (22). In addition, HDAC inhibitors have been revealed to exhibit synergy with numerous anti-cancer agents, including cytotoxic agents such as gemcitabine, cisplatin, etoposide, paclitaxel and doxorubicin (18). However, the development of resistance to chemotherapy is a major impediment for any novel cancer therapy. Despite HDAC inhibitors being a novel class of potent anticancer drug, previous studies have revealed that exposure of cancer cells to HDAC inhibitors can lead to broad-spectrum anticancer MDR, resulting in cells that are resistant to numerous structurally and functionally unrelated drugs (27). One of the phenotypes of MDR is the upregulation of ABC transport proteins, which decrease the level of intracellular chemotherapeutic drugs in an energy-dependent manner (25,26). ABC transporters mainly include P-gp, BCRP and MRPs, which are coded for by MDR1, ABCG2 and ABCC, respectively. P-gp, the most well-known ABC transporter, is a membrane-bound transporter that extrudes natural toxins and drug metabolites, as well as anticancer drugs, across the plasma membrane, which in turn leads to drug resistance in various cell lines. Previous studies have revealed the induction of P-gp in human and murine cells exposed to HDAC inhibitors, including TSA, VPA and apicidin (7,26,28,29). The protein expression of BCRP and certain members of the MRP family is also elevated in cancer cells following treatment with HDAC inhibitors (26,30,31). However, there are also controversial reports suggesting that HDAC inhibitors can overcome MDR through reducing the expression of ABC transporters (32).

In the following section of this review, the collated information is summarized and analyzed, with the aim of clarifying the potential association between known HDAC inhibitors and MDR following treatment, and particularly the role of the ABC transporters in this process.

### Cyclic tetrapeptides

Depsipeptide (FK228) and P-gp, BCRP and MRP1. FK228 (Istodax), also known as FR901228, depsipeptide or romidepsin, was developed and approved by the FDA for the treatment of CTCL in 2009 (5,8,30,31,33,34). FK228 acts as a prodrug, with the disulphide bond undergoing reduction within the cell to release Zn^2+^, which binds thiol in the binding pocket of HDAC (14). As an HDAC inhibitor, the potency of FK228 is at a nanomolar concentration (6).

The development of drug resistance is a notable concern with any chemotherapy agent, and FK228 is not an exception (8). Previous studies have reported that FK228 is a substrate for P-gp, which is the most characterized ABC transporter responsible for MDR (30,33). FK228 is, to date, the only HDAC inhibitor known to be a substrate for P-gp (35). Yamada *et al* (35) further demonstrated that continued exposure to FK228 induced P-gp expression and the overexpression of P-gp was reversed upon the removal of FK228 (8).

In addition to being a substrate for P-gp, Dean *et al* identified that FK228 was the substrate of MRP1, which was found to be involved in the mechanism causing MDR following treatment with FK228 (36). MRP1 is known to participate in the transport of glutathione conjugates of numerous toxic compounds (30). Nevertheless, Xiao *et al* found that the mechanism of MRP1 leading to MDR did not result from the upregulation of ABCC1 expression, indicating that the expression of P-gp and MRP1 may be controlled by different mechanisms (30). Confusingly, MRP1 was not induced in all the examined cell lines when increasing exposure to FK228, including four FK228-resistant cell lines; IGROV1, MCF7, K562 and human colorectal adenocarcinoma HCT-15 cells, (33). This suggested that FK228-induced MDR was likely to be cell line-dependent.

Similarly, To *et al* found that BCRP, another ABC transporter that is encoded by ABCG2, could contribute to drug resistance following continuous treatment with FK228 (31). The activation of BCRP by FK228 at the transcriptional and protein levels has been reported in renal and colon cancer cell lines (37). Nevertheless, MDR1 was upregulated following FK228 treatment in all the studied cell lines, whereas the expression of ABCG2 was increased only in the cell lines in which the repressive mark, Me3-K9 H3 (trimethylated histone H3 lysine 9), was removed, HDAC was disassociated and then RNA polymerase II was recruited to the ABCG2 promoter (31). The FK228-induced expression of ABCG2 was cell-specific due to dynamic changes in chromatin structure.

#### Apicidin and P-gp

Apicidin [cyclo(*N*-*O*-methyl-L-tryptophanyl-L-isoleucinyl-D-pipecolinyl-L-amino-8-oxodecanoyl)], a cyclic tetrapeptide that can be isolated from the culture of Fusarium pallidoroseum, is a novel potent HDAC inhibitor that selectively inhibits HDACs 1 and 3, but not HDAC8 (38,39). Apicidin has demonstrated a broad spectrum of anti-proliferative activity in various cancer cell lines, including leukemia, and cervical, gastric, breast and endometrial cancer cell lines (40,41). In addition to solid cancer cell lines, apicidin exhibits effective anticancer potential in human acute promyelocytic leukemia cells (42).

Two separate studies have each reported that apicidin induces MDR (7,43). Apicidin resistance to the apoptotic potential of paclitaxel was associated with the induction of P-gp expression, including the mRNA and protein expression level in HeLa cells (7). There was evidence to suggest that apicidin did not alter the cytosine-phosphate-guanine (CpG) methylation status of the P-gp promoter region, which is significant, as hypermethylation of CpG is always associated with low expression of P-gp in various cell lines and tissues obtained from patients (44). Exposure to apicidin leads to chemoresistance of cancer cells through the induction of P-gp expression. By contrast, in human glioblastoma A172 and U87 cell lines or human oral cancer KB cells, continuous exposure to apicidin does not increase P-gp expression or reduce paclitaxel-induced cytotoxicity, indicating that P-gp is induced by apicidin in a cell type-specific manner (28,43,44). These studies speculated that the cell type-specific induction of P-gp expression may rely on phosphatidylinositol 3-kinase activity in addition to the CpG methylation status of the promoter (43). However, this exact mechanism has yet to be clearly elucidated.

### Hydroxamic acids

#### SAHA and MRP2

Vorinostat [Zolinza; SAHA] is a novel hydroxamate structure HDAC inhibitor and was the most established HDAC inhibitor approved by the FDA for the treatment of CTCL in 2006 (18,45). SAHA was found to upregulate the cyclin-dependent kinase inhibitor p21^WAF1/CIP1^, inhibit tumor cell growth and induce apoptosis (12,46).

As a potent inhibitor, SAHA exhibits a good bioavailability and low toxic effects (6,14). However, the antineoplastic activity of SAHA, similar to FK228, was found to be a potential inducer of HDAC inhibitor resistance (30,47). Fedier *et al* (47) demonstrated that SAHA induced drug resistance following continuous treatment in a MDR1-independent manner, which is noteworthy as SAHA is not a substrate for MDR1 (30,48). However, another study by Kim *et al* confirmed that SAHA did not affect MDR1 expression (32). Kim *et al* reported that SAHA may overcome MDR through a specific downregulation of MRP2 in MDR cancer cell lines following exposure to SAHA. When paclitaxel was used in combination with SAHA, the downregulation of MRP2 led to an increase in G_2_/M arrest and apoptosis, indicating that SAHA may be useful for MDR cancer treatment as a potent HDAC inhibitor (32). At present, the exact molecular mechanism for the specific downregulation of MRP2 requires further investigation.

Currently, numerous studies investigate the role and mechanism of SAHA, particularly its role in cancer treatment, but only a few studies investigate MDR, leading to less knowledge of the mechanism that induces or overcomes resistance when using SAHA. In the present review, one study investigating MDR following the use of SAHA reported that SAHA can induce cross-resistance to the hydroxamate-class and to the aliphatic acid-class (VPA) HDAC inhibitors, but not to the benzamide-class (49). It has also been reported that no acquisition of resistance by SAHA is observed in HeLa cells, while acquisition of resistance can be observed in HCT116 cells, which may be due to a type of cell-specific induction (49). Studies also report that SAHA may have a different mechanism for the induction of resistance, unlike FK228, and that, according to various experiment results, SAHA can not only induce MDR, but also overcome MDR, which appears to be contradictory (7,30,32,43,47,48). Additional studies that investigate the issue of SAHA-induced MDR are required.

#### TSA and P-gp

TSA is a natural hydroxamate HDAC-inhibitor with a structure similar to that of SAHA. TSA has been reported to be an non-selective inhibitor at low micromolar concentrations and to exert synergistic effects in combination with paclitaxel (1,2,14). However, TSA has also been found to be cytotoxic in a broad range of cells, limiting the clinical use of TSA (50). Yatouji *et al* described the fact that TSA induced an increase in the level of acetylated H4, which was associated with the decondensation of chromatin and an increase in the gene expression of MDR1, resulting in MDR (28). Nevertheless, similar to SAHA, TSA is able to downregulate the expression of MRP2 in multidrug-resistant cancer cells. Kim *et al* hypothesized that numerous HDAC inhibitors could strengthen chemosensitization in multidrug-resistant cancer cells (32). This type of HDAC inhibitor may be used to overcome MDR in cancer cells.

### Short-chain fatty acids

#### VPA, P-gp and MRP2

VPA has been used for numerous years to treat epilepsy and bipolar disorders, and it is now undergoing phase I/II-stage clinical trials as a potent HDAC inhibitor against leukemia, myelodysplasia and cervical cancer (14). Similarly, the findings on the effect of VPA on drug resistance are controversial. It was reported that VPA induced resistance mainly through increased P-gp expression and function in a time-dependent manner in human tumor cell lines and in rat liver, but that it is not a substrate of P-gp (29,51). By contrast, Fedier *et al* revealed that VPA led to MDR in a MDR1-independent manner, by reducing the expression of MRP2 (47). The disparity may be explained by a difference in the human colon cell lines used in the studies investigating VPA drug resistance. In particular, the cell line of the former study was HCT, while that of the latter study was SW620, indicating that the VPA-induced drug resistance may vary between different cell lines and tissues, as reported by Dedes *et al* (49).

BT and BCRP. BT, which is produced through intestinal flora fermentation of dietary fiber, plays an important role in colorectal carcinogenesis (52). BT has fostered the most attention as an HDAC inhibitor and can also induce MDR through the activation of ABC transporters. Previous studies have revealed that BT induces the expression of MDR1 (53–56). Another study has indicated that, similar to TSA, sodium BT led to MDR1 upregulation in drug-sensitive H69WT cells, but downregulation in drug-resistant H69VP cells (57). Hauswald *et al* noted that phenylbutyrate, as a type of BT, can induce P-gp and BCRP expression (26). By contrast, Gonçalves *et al (58)* found no induction of MDR1 and MRPs in BT-treated Caco 2 cells. Despite the conflicting findings on the effect of BT on MDR1 expression, the study confirmed the elevated expression of BCRP following BT treatment, and also demonstrated that BT was a substrate of rat and human BCRP. Therefore, the inhibition of BCRP can significantly potentiate the effect of BT on cell proliferation and suppress BT-induced drug resistance due to the overexpression of BCRP (58).

### Benzamides

Entinostat (MS-275) is a benzamide that is well known as a selective class I inhibitor (4,22). It has been revealed that MS-275 induces differentiation in AML cells (22). MS-275 has demonstrated potent anti-tumor activities through increasing the levels of p21 in a concentration-dependent manner and is currently in phase I or II clinical trials (18,32). One study found that MS-275 did not affect the expression of MDR1 and BCRP, and that it slightly decreased the level of MRP2 mRNA expression, but not the protein level (32). However, the finding that ABC transporters are activated by the treatment of MS-275 requires further verification.

## 5. Other mechanisms of HDAC inhibitor-induced MDR

### Nuclear receptors

It is well known that ABC transporters are regulated by nuclear factors. To further elucidate the mechanism of HDAC inhibitor-induced resistance, a few studies identified that certain HDAC inhibitors affected nuclear factors in cancer cell lines. Cerveny *et al* (59) noted that VPA could activate the constitutive androstane receptor (CAR) pathway, inducing MDR1 gene expression (60). VPA upregulated CAR/retinoid X receptor heterodimer binding to the direct repeat 4-responsive element of MDR1 genes. CAR belongs to the orphan nuclear receptor superfamily, which also includes the pregnane X receptor, liver X receptor (LXR) and farnesoid X receptor (FXR). The activation of cytosolic CAR contributes to its dissociation from its co-chaperone partners, CAR retention protein and heat shock protein 90, and increases the transcription of target genes (61). Another study illustrated that apicidin downregulated estrogen receptor α expression in MCF-7 cells (62), while Korkmaz *et al* found that HDAC inhibitors, including TSA and BT, could potentiate androgen receptor transcriptional activity (63). Other nuclear factors, such as LXR and FXR, can also regulate the expression of ABC transporters (64–67). However, there are relatively less relevant studies into HDAC inhibitor-induced drug resistance involving nuclear receptors.

### Other factors

In addition to ABC transporters and nuclear factors, other associated mechanisms can also induce MDR. Robey *et al* summarized possible causes for MDR following the use of HDAC inhibitors, including the inducement of cell cycle protein p21, the upregulation of thioredoxin levels and the activation of NF-κB, affected apoptosis-associated proteins or signaling proteins (68). ABC transporters and cytochrome P450 (CYP) enzymes are well-known targets of nuclear factors. Certain studies revealed that CYP enzymes were induced by HDAC inhibitors in the human mammary carcinoma-derived MCF-7 and HeLa cell lines (69,70). Cerveny *et al* and Takizawa *et al* also reported that HDAC inhibitors could induce CYP enzymes involving nuclear factors (59,71). Thus, CYP enzymes may be involved in HDAC inhibitor-induced MDR. In addition, cancer stem cells (CSCs) are believed to contribute to cancer initiation, progression, metastasis, recurrence and drug resistance (72). However, there is still a lack of studies investigating the association between HDAC inhibitors and CSCs leading to resistance.

At present, numerous HDAC inhibitors are at various stages of clinical trials, while SAHA and FK228 have been approved by the FDA (6). However, HDAC inhibitors can induce MDR, mainly through the effect on ABC transporters, which causes cancer treatment to exhibit a poor prognosis. It has been found that various HDAC inhibitors may exert diverse impacts on ABC transporters. Certain HDAC inhibitors, including TSA, FK228 and apicidin, can induce MDR in cancer cell lines through upregulation of specific ABC transporters, such as P-gp. By contrast, HDAC inhibitors, such as SAHA and VPA, can overcome MDR by downregulating MRP2 and other transporters. Furthermore, in various cancer cell lines, the same HDAC inhibitor may have opposite effects on the expression of ABC transporters (31). Currently, the exact mechanism of HDAC inhibitor-induced MDR in various cell lines has yet to be elucidated. Additional studies investigating the association between HDAC inhibitors and ABC transporters are required.

At present, it has been suggested that HDAC inhibitors should be applied in combination with inhibitors of ABC transporters (7,30). microRNAs, other than those targeting HDAC, are becoming a novel therapeutic starting point to optimize therapy and overcome HDAC inhibitor-induced pharmacoresistance (73).

In summary, a clear understanding with regard to HDAC inhibitors as anticancer agents, and also the issue of MDR whilst treating cancer, should be developed. The upregulation of ABC transporters, leading to MDR subsequent to treatment with HDAC inhibitors, should be taken into consideration, particularly when HDAC inhibitors are used to pretreat patients receiving drugs that are substrates of ABC transporters, and in particular, P-gp substrates.

## Abbreviations

HAT: histone acetyl transferases
HDAC: histone deacetylases
MDR: multidrug resistance
ABC: adenosine triphosphate-binding cassette
P-gp: P-glycoprotein
BCRP: breast cancer resistance protein
MRP: multidrug resistance protein
SAHA: suberoylanilide hydroxamic acid
TSA: trichostatin A
VPA: valproic acid
BT: butyrate
CYP: cytochrome P450
CAR: constitutive androstane receptor
LXR: liver X receptor
FXR: farnesoid X receptor
